# ZnO Nanoparticles as an Efficient, Heterogeneous, Reusable, and Ecofriendly Catalyst for Four-Component One-Pot Green Synthesis of Pyranopyrazole Derivatives in Water

**DOI:** 10.1155/2013/680671

**Published:** 2013-10-24

**Authors:** Harshita Sachdeva, Rekha Saroj

**Affiliations:** Department of Chemistry, Faculty of Engineering and Technology, Mody Institute of Technology and Science, Lakshmangarh, Sikar, Rajasthan 332311, India

## Abstract

An extremely efficient catalytic protocol for the synthesis of a series of pyranopyrazole derivatives developed in a one-pot four-component approach in the presence of ZnO nanoparticles as heterogeneous catalyst using water as a green solvent is reported. Greenness of the process is well instituted as water is exploited both as reaction media and medium for synthesis of catalyst. The ZnO nanoparticles exhibited excellent catalytic activity, and the proposed methodology is capable of providing the desired products in good yield (85–90%) and short reaction time. After reaction course, ZnO nanoparticles can be recycled and reused without any apparent loss of activity which makes this process cost effective and hence ecofriendly. All the synthesized compounds have been characterized on the basis of elemental analysis, IR, ^1^H NMR, and ^13^C NMR spectral studies.

## 1. Introduction

 Multicomponent reactions (MCRs) occupy an interesting position in organic synthesis because of their atom economy, simple procedures, and convergent character [1–3]. Applications of MCRs in drug discovery, material sciences, natural product synthesis, and ligand and biological probe preparations further demonstrate the power of this reaction [4, 5].

Catalysis has played a vital role in the success of the industry [6]. The use of transition-metal nanoparticles in catalysis is crucial as they mimic metal surface activation and catalysis at the nanoscale and thereby bring selectivity and efficiency to heterogeneous catalysis [7–14]. Among transition-metal nanoparticles, ZnO nanoparticles have been of considerable interest because of the role of ZnO in solar cells, catalysts, antibacterial materials, gas sensors, luminescent materials, and photocatalyst [15]. The recent literature survey reveals that nano-ZnO as heterogeneous catalyst has received considerable attention because it is inexpensive, nontoxic catalyst and has environmental advantages, that is, minimum execution time, low corrosion, waste minimization, recycling of the catalyst, easy transport, and disposal of the catalyst.

In recent years, in biological field, the potential utility of ZnO nanoparticle in the treatment of cancer has been reported by many researchers. Owing to numerous advantages associated with this ecofriendly nature, it has been explored as a powerful catalyst for several organic transformations [16–21] such as Mannich reaction, and the Knoevenagel condensation reaction, in the synthesis of coumarins, quinolines, polyhydroquinoline, 2,3-disubstituted quinalolin-4(1H)-ones, and benzimidazole.

Pyrazole derivatives constitute an interesting class of heterocycles due to their synthetic versatility and effective biological activities [22–28]. Further, pyrano[2,3-*c*]pyrazoles constitute one of the privileged heterocyclic scaffolds known to exhibit important biological activities [29–32]. Nowadays, there has been increasing interest in the development of nonhazardous alternatives such as water-mediated syntheses, multicomponent reactions, and reusable heterogeneous catalysts for the sustainable development of chemical enterprise. Although numerous methods to achieve pyranopyrazoles are known [33–44], simple, environmentally benign approaches are still demanded.

**Scheme 1:**
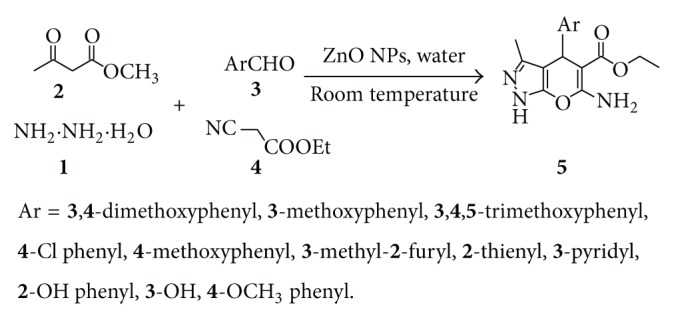


 Hence, in continuation of our work to develop ecofriendly techniques for heterocyclic synthesis [45–47], an attempt has been made to synthesize pyranopyrazole derivatives by the reaction of hydrazine hydrate, methyl acetoacetate, substituted aromatic aldehydes, and ethyl cyanoacetate in water using ZnO nanoparticles as catalyst at room temperature (25°C) under the framework of green chemistry (Scheme 1). 

The process described here offers rapid facile one-pot synthesis of pyranopyrazole derivatives using easily recyclable ZnO nanoparticles. This process is cost effective and eco-friendly as it is one-pot synthesis with easy work-up and does not require harsh reagents. To the best of our knowledge, there is no report available in the literature describing the use of ZnO nanoparticles as catalysts for the synthesis of pyranopyrazole carboxyethylester derivatives. The effectiveness of the process was studied by comparing the results obtained with and without catalyst under normal conditions. 

## 2. Experimental

### 2.1. General

 Reagents and solvents were obtained from commercial sources and used without further purification. Melting points were determined on a Toshniwal apparatus. The spectral analyses of synthesized compounds have been carried out at SAIF, Punjab University, Chandigarh. Purity of all compounds was checked by TLC using “G” coated glass plates and benzene: ethyl acetate (8 : 2) as eluent. IR spectra were recorded in KBr on a Perkin Elmer Infrared RXI FTIR spectrophotometer, and ^1^H NMR spectra were recorded on Bruker Avance II 400 NMR spectrometer using DMSO-d_6_ and CDCl_3_ as solvent and tetramethylsilane (TMS) as internal reference standard. The obtained products were identified from their spectral (^1^H NMR, ^13^C NMR, and IR) data. The microwave-assisted reactions were carried out in a Catalysts Systems Scientific Multimode MW oven attached with a magnetic stirrer and reflux condenser, operating at 700 W generating 2450 MHz frequency. 

### 2.2. General Procedure for the Synthesis of ZnO Nanoparticles in Water

ZnO nanoparticles were synthesized by two different methods.

#### 2.2.1. Method A

ZnO nanorods are prepared according to a literature method developed by Pacholski et al. [48] with some modification. Firstly, zinc acetate (Zn(Ac)_2_, 2.4 g) and 126 mL of water were added into a round bottom flask. The solution was heated to 60°C with magnetic stirring. Potassium hydroxide (KOH, 1.2 g) was dissolved into 70 mL of water as the stock solution that is dropped into the flask within 10–15 min. At a constant temperature of 60°C, it takes 2 hrs and 15 min. A small amount of water was found helpful to increase the ZnO nanocrystal growth rate. To grow the nanorods, the solution is condensed to about 10–15 mL. This was found helpful before further heating to decrease the growth time of the nanorods. Then it is reheated for another 5 hrs before stopping the heating and stirring. The upper fraction of the solution is removed after 30 min. Water (50 mL) is added to the solution and stirred for 5 min. The upper fraction of the solution is discarded again after 30 min. This process is repeated twice. After being dried under vacuum, ZnO nanoparticles were obtained (yield: 85%.) 

#### 2.2.2. Method B

Zinc acetate and hydrazine hydrate were mixed in a molar ratio of 1 : 4 in water under stirring. Hydrazine readily reacted with zinc acetate to form a slurry-like precipitate of the hybrid complex between them. The stirring of the slurry was continued for 15 min, and then the mixture was subjected to microwave irradiation at 150 W microwave power for 10 min. The slurry became clear with a white precipitate at the bottom. The precipitate was filtered off, washed with absolute ethanol and distilled water several times and then dried in vacuum at 60°C for 4 hrs (yield: 78%.)

### 2.3. Synthesis of 3-Methylpyrano[2,3-*c*]pyrazole Derivatives (**5a–j**)

**Figure 1:**
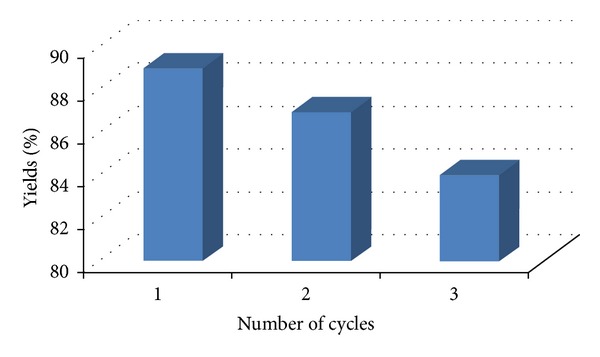
Recyclability of ZnO nanoparticles.

A mixture of hydrazine hydrate **(1)** (1 mmol), methyl acetoacetate **(2)** (1 mmol), substituted aromatic aldehyde **(3)** (1 mmol), ethylcyano acetate **(4)** (1 mmol), and ZnO nanoparticle (9 mol%) in water (2 mL) was magnetically stirred at room temperature (25°C) for 55–60 min. Progress of the reaction was monitored by TLC. After completion of the reaction, the resulting solidified mixture was diluted with ethyl acetate (5 mL), the catalyst was separated, and the reaction mixture was subjected for solvent-extraction again using ethyl acetate (3 × 10 mL). Thus obtained portion of organic layer (ethyl acetate) was concentrated on rotary evaporator under reduced pressure to achieve the desired product. This crude product was purified by recrystallization from ethanol. Results are given in Table 1. ZnO nanoparticles thus obtained were washed with methanol and could be reused for the next cycle. The catalyst retained optimum activity till three cycles after which drop in yield was observed (Figure 1). 

*Synthesis of* **5e** *by Conventional *Δ* Heating.* For comparison's sake, compound **5e** was also synthesized by conventional Δ heating. An equimolar mixture of hydrazine hydrate **(1)** (1 mmol), methyl acetoacetate **(2)** (1 mmol), 4-methoxy benzaldehyde **(3)** (1 mmol), ethylcyano acetate **(4)** (1 mmol), and ZnO nanoparticles (9 mol%) in water (2 mL) was refluxed for 40 min. Progress of the reaction was monitored by TLC using ethyl acetate : benzene = 2 : 8 as eluent. After completion of the reaction, the mixture was subjected to solvent-extraction using ethyl acetate, and obtained portion of organic layer was concentrated on rotary evaporator under reduced pressure to achieve the desired product. This crude product was purified by recrystallization from ethanol. The comparative results obtained by different methods for the synthesis of compound **5e** are given in Table 4.

### 2.4. Regeneration of Catalyst

To examine the reusability, the catalyst was recovered by filtration from the reaction mixture after dilution with ethyl acetate, washed with methanol, and reused as such for subsequent experiments (up to three cycles) under similar reaction conditions. The observed fact that yields of the product remained comparable in these experiments (Figure 1) established the recyclability and reusability of the catalyst without any significant loss of activity.

## 3. Results and Discussion

 An environ-economic synthesis of ethyl-6-amino-1, 4-dihydro-3-methyl-4-substituted  pyrano[2,3-*c*]pyrazole-5-carboxylate derivatives **(5a–j)** is carried out by the reaction of hydrazine hydrate **(1)**, methylacetoacetate **(2)**, substituted aromatic aldehydes **(3)**, and ethylcyano acetate **(4)** in the presence of catalytic amount of ZnO nanoparticle as catalyst under stirring at room temperature 25°C in the presence of water (Scheme 1) (Table 1). Reaction of methylacetoacetate, hydrazine hydrate, 4-methoxy benzaldehyde, and ethylcyanoacetate **(5e)** was chosen as the model substrate to optimize reaction condition including type of catalyst and concentration of catalyst. 

We have extensively studied the reaction using various catalysts such as alum, Montmorillonite-K10 clay, P_2_O_5_, acidic alumina, silica, Montmorillonite-KSF clay, glacial acetic acid, and ZnO nanoparticles (Table 2). The results showed that ZnO nanoparticle provided the highest yield (89%). The effect of solvents was also examined for the above reaction, and the results indicate that solvents affected the efficiency of the reaction. Yields were poor in ethanol and methanol under stirring at room temperature. However, the best results were obtained in the presence of water (Table 3). In order to confirm the effective involvement of ZnO nanoparticle during this transformation, we carried out the model reaction without any catalyst. In the absence of ZnO nanoparticle, the reaction was incomplete even after 8 hrs of stirring at room temperature and 6 hrs of conventional Δ heating (Table 4). Traces of product were observed on TLC.

Encouraged by these results, we have extended this reaction to variously substituted aromatic aldehydes under similar conditions using ZnO nanoparticle as a catalyst to furnish the respective pyranopyrazole derivatives in excellent yields (85–90%) without the formation of any side products. Further, we have emphasized the amount of ZnO nanoparticle to be used in this reaction. We found that the yields were obviously affected by the amount of ZnO nanoparticles loaded. When 3, 6, 9, and 12 mol% of ZnO nanoparticles was used, the yields were 75%, 82.06%, 89%, and 89%, respectively. Therefore, 9 mol% of ZnO nano particles were sufficient to push the reaction forward, and, further, increasing the amount of ZnO nanoparticles did not increase the yields (Table 5).

The above results indicate that ZnO nanoparticle was essential in the reaction and the best results were obtained when the reaction was carried out with 9 mol% of ZnO nanoparticles at room temperature.

The proposed mechanism for the formation of the product would be as follows. The ZnO nanoparticle facilitates the Knoevenagel type coupling through Lewis acid sites (Zn^+2^) coordinated to the oxygen of carbonyl groups of methylacetoacetate. On the other hand, ZnO nanoparticles can activate ethylcyanoacetate so that deprotonation of the C–H bond occurs in the presence of Lewis basic sites (O^−2^). As a result, the formation of pyranopyrazole derivatives proceeds by activation of reactants through both Lewis acids and basic sites of ZnO nanoparticles. The reaction occurs via initial formation of arylidene ethylcyanoacetate by the Knoevenagel condensation between aromatic aldehyde and ethyl cyanoacetate and pyrazolone by the reaction of methyl acetoacetate and hydrazine hydrate. Finally, the Michael addition of pyrazolone to arylidene ethylcyanoacetate followed by cyclization and tautomerization yields pyranopyrazole. 

The synthesis of ZnO nanoparticles was carried out in distilled water for its inherent advantages as it is simple, cost effective, environmentally benign, and easily scaled up for large scale synthesis, and in this method there is no need to use high pressure, high temperature, and toxic chemicals. Additionally, water served as a suitable solvent for the current transformation as well.

Reusability (and hence recyclability) is one of the important properties of this catalyst. The catalyst could be recycled easily, simply by solvent extraction of the product from the reaction mixture using ethyl acetate. The catalyst retained optimum activity till three cycles after which drop in yield was observed (Figure 1). A comparison of efficiency of catalytic activity of ZnO nanoparticles with other catalysts is presented in Table 2. The results show that this method is superior to other methods in terms of yield and reaction time.

**Figure 2:**
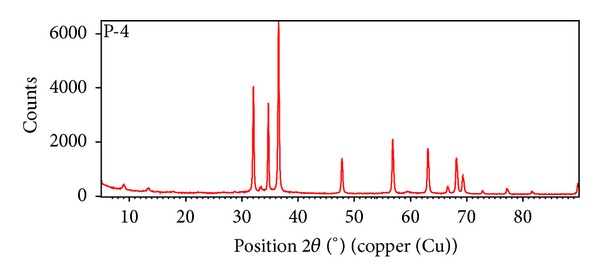
XRD Pattern of ZnO nanoparticles.

 The nanostructure of ZnO nanoparticle has been studied at room temperature by using X-ray diffraction pattern.  Figure 2 shows XRD pattern of ZnO nanoparticles. The particle size was calculated from X-ray diffraction images of ZnO powders using Scherrer formula as follows:

(1) $$\begin{array}{c} D=\frac{K\lambda }{\beta \cos\theta }, \end{array}$$

where *D* is the average particle size perpendicular to the reflecting planes, *λ* is the X-ray wavelength, *β* is the full width at half maximum (FWHM), and *θ* is the diffraction angle. The average size of ZnO nanoparticles obtained from the XRD is about 5.1 nm, using the Scherrer formula.

The spectroscopic characterization data of the synthesized compounds are given below.

*Ethyl-6-amino-1,4-dihydro-4-(3,4-dimethoxyphenyl)-3-methylpyrano[2,3-c]pyrazole-5-carboxylate* **(5a)**. M.P. 135°C; IR (KBr): 3411, 3355, 3082, 2943, 1729, 1142 cm^−1^; ^1^H NMR (DMSO-d_6_): 1.30 (t, 3H, CH_3_), 2.79 (s, 3H, CH_3_), 3.73 (s, 6H, 2 × OCH_3_) 4.19 (q, 2H, CH_2_), 4.74 (s, 1H, CH), 6.46–6.54 (m, 3H, ArH), 7.06 (s, 2H, NH_2_), 12.08 (s, 1H, NH) ppm. ^13^C NMR (400 MHz, DMSO): 10.34, 13.66, 38.84, 55.64, 61.80, 78.74, 114.12–132.38, 140.06, 146.8, 160.32, 164.28 ppm. Anal. calcd for C_18_H_21_N_3_O_5_: C, 60.16; H, 5.89; N, 11.69. Found: C, 60.00; H, 5.91; N, 11.67.

*Ethyl-6-amino-1,4-dihydro-4-(3-methoxyphenyl)-3-methylpyrano[2,3-c]pyrazole-5-carboxylate* **(5b)**. M.P. 120°C; IR (KBr): 3419, 3351, 3100, 2933, 1719, 1158 cm^−1^; ^1^H NMR (DMSO-d_6_): 1.31 (t, 3H, CH_3_), 2.78 (s, 3H, CH_3_), 3.72 (s, 3H, OCH_3_) 4.20 (q, 2H, CH_2_), 4.72 (s, 1H, CH), 6.48–7.03 (m, 4H, ArH), 7.07 (s, 2H, NH_2_), 12.09 (s, 1H, NH) ppm. ^13^C NMR (400 MHz, DMSO): 10.36, 13.64, 38.82, 55.62, 61.88, 78.78, 111.32–132.32, 140.02, 159.12, 160.38, 164.12 ppm. Anal. calcd for C_17_H_19_N_3_O_4_: C, 62.00; H, 5.81; N, 12.76. Found: C, 62.17; H, 5.79; N, 12.74.

*Ethyl-6-amino-1,4-dihydro-4-(3,4,5-trimethoxyphenyl)-3-methylpyrano[2,3-c]pyrazole-5-carboxylate* **(5c)**. M.P. 160°C; IR (KBr): 3410, 3359, 3092, 2949, 1722, 1153 cm^−1^; ^1^H NMR (DMSO-d_6_): 1.30 (t, 3H, CH_3_), 2.50 (s, 3H, CH_3_), 3.86 (s, 9H, 3 × OCH_3_) 4.18 (q, 2H, CH_2_), 4.70 (s, 1H, CH), 6.02–6.12 (m, 2H, ArH), 7.04 (s, 2H, NH_2_), 12.03 (s, 1H, NH) ppm. ^13^C NMR (400 MHz, DMSO): 10.32, 13.62, 38.86, 55.66, 61.82, 78.76, 105.38–132.32, 140.02, 152.12, 160.38, 164.28 ppm. Anal. calcd for C_19_H_23_N_3_O_6_: C, 58.60; H, 5.95; N, 10.79. Found: C, 58.81; H, 5.94; N, 10.80.

*Ethyl-6-amino-4-(4-chlorophenyl)-1,4-dihydro-3-methylpyrano[2,3-c]pyrazole-5-carboxylate* **(5d)**. M.P. 140°C; IR (KBr): 3411, 3355, 3082, 2943, 1729, 848 cm^−1^; ^1^H NMR (DMSO-d_6_): 1.27 (t, 3H, CH_3_), 2.77 (s, 3H, CH_3_), 4.22 (q, 2H, CH_2_), 4.71 (s, 1H, CH), 7.02–7.15 (m, 4H, ArH), 7.02 (s, 2H, NH_2_), 12.05 (s, 1H, NH) ppm. ^13^C NMR (400 MHz, DMSO): 10.32, 13.68, 38.82, 61.88, 78.76, 128.82–130.56, 116.04–132.32, 131.30, 140.02, 160.32, 164.22 ppm. Anal. calcd for C_16_H_16_ClN_3_O_3_: C, 57.58; H, 4.83; N, 12.59. Found: C, 57.76; H, 4.85; N, 12.58.

*Ethyl-6-amino-1,4-dihydro-4-(4-methoxyphenyl)-3-methylpyrano[2,3-c]pyrazole-5-carboxylate* **(5e)**. M.P. 130°C; IR (KBr): 3415, 3350, 3095, 2963, 1739, 1152 cm^−1^; ^1^H NMR (DMSO-d_6_): 1.28 (t, 3H, CH_3_), 2.75 (s, 3H, CH_3_), 3.71 (s, 3H, OCH_3_) 4.17 (q, 2H, CH_2_), 4.73 (s, 1H, CH), 6.65–6.95 (m, 4H, ArH), 7.03 (s, 2H, NH_2_), 12.02 (s, 1H, NH) ppm. ^13^C NMR (400 MHz, DMSO): 10.34, 13.62, 38.82, 55.68, 61.86, 78.72, 114.22–132.38, 140.04, 156.22, 159.44, 160.36, 164.24 ppm. Anal. calcd for C_17_H_19_N_3_O_4_: C, 62.00; H, 5.81; N, 12.76. Found: C, 62.19; H, 5.79; N, 12.75.

*Ethyl-6-amino-1,4-dihydro-3-methyl-4-(5-methylfuran-2-yl)pyrano[2,3-c]pyrazole-5-carboxylate* **(5f)**. M.P. 142°C; IR (KBr): 3427, 3365, 3077, 2949, 1739, 1166 cm^−1^; ^1^H NMR (DMSO-d_6_): 1.22 (t, 3H, CH_3_), 2.17 (s, 3H, CH_3_), 2.75 (s, 3H, CH_3_), 4.17 (q, 2H, CH_2_), 4.73 (s, 1H, CH), 6.02–6.28 (m, 2H, ArH), 6.65–6.95 (m, 4H, ArH), 7.03 (s, 2H, NH_2_), 12.02 (s, 1H, NH) ppm. ^13^C NMR (400 MHz, DMSO): 10.32, 13.64, 14.2, 38.72, 61.83, 78.72, 114.22–132.38, 140.04, 156.20, 159.40, 160.33, 164.20 ppm. Anal. calcd for C_15_H_17_N_3_O_4_: C, 59.40; H, 5.65; N, 13.85. Found: C, 59.57; H, 5.63; N, 13.86.

*Ethyl-6-amino-1,4-dihydro-3-methyl-4-(thiophen-2-yl)pyrano[2,3-c]pyrazole-5-carboxylate* **(5g)**. M.P. 115°C; IR (KBr): 3416, 3352, 3099, 2940, 1722, 1279 cm^−1^; ^1^H NMR (DMSO-d_6_): 1.24 (t, 3H, CH_3_), 2.72 (s, 3H, CH_3_), 4.22 (q, 2H, CH_2_), 4.78 (s, 1H, CH), 6.60–6.91 (m, 4H, ArH), 6.69–6.98 (m, 3H, ArH), 7.08 (s, 2H, NH_2_), 12.04 (s, 1H, NH) ppm. ^13^C NMR (400 MHz, DMSO): 10.38, 13.60, 38.80, 61.86, 78.72, 114.22–132.38, 123.6–139.4, 140.04, 156.22, 159.44, 160.36, 164.24 ppm. Anal. calcd for C_14_H_15_N_3_O_3_S: C, 55.07; H, 4.95; N,13.76. Found: C, 55.27; H, 4.93; N, 13.77.

*Ethyl-6-amino-1,4-dihydro-3-methyl-4-(pyridin-3-yl)pyrano[2,3-c]pyrazole-5-carboxylate* **(5h)**. M.P. 125°C; IR (KBr): 3431, 3345, 3089, 2953, 1722, 1520 cm^−1^; ^1^H NMR (DMSO-d_6_): 1.28 (t, 3H, CH_3_), 2.75 (s, 3H, CH_3_), 4.17 (q, 2H, CH_2_), 4.73 (s, 1H, CH), 6.65–6.95 (m, 4H, ArH), 7.08 (s, 2H, NH_2_), 7.29–8.57 (m, 4H, ArH), (12.06 (s, 1H, NH) ppm. ^13^C NMR (400 MHz, DMSO): 10.34, 13.62, 38.82, 61.80, 78.78, 114.20–132.20, 123.34–152.56, 140.02, 156.20, 159.32, 160.32, 164.21 ppm. Anal. Calcd for C_15_H_16_N_4_O_3_: C, 59.99; H, 5.37; N, 18.66. Found: C, 59.81; H, 5.35; N, 18.65.

*Ethyl-6-amino-1,4-dihydro-4-(2-hydroxyphenyl)-3-methylpyrano[2,3-c]pyrazole-5-carboxylate* **(5i)**. M.P. 143°C; IR (KBr): 3410, 3389, 3335, 3072, 2931, 1734 cm^−1^; ^1^H NMR (DMSO-d_6_): 1.30 (t, 3H, CH_3_), 2.79 (s, 3H, CH_3_), 4.19 (q, 2H, CH_2_), 4.74 (s, 1H, CH), 5.02 (s, 1H, OH), 6.46–6.54 (m, 4H, ArH), 7.06 (s, 2H, NH_2_), 12.08 (s, 1H, NH) ppm. ^13^C NMR (400 MHz, DMSO): 10.38, 13.64, 38.82, 55.68, 61.82, 78.78, 115.44–132.34, 140.06, 145.14, 160.44, 164.28 ppm. Anal. calcd for C_16_H_17_N_3_O_4_: C, 60.94; H, 5.43; N, 13.33. Found: C, 60.73; H, 5.41; N, 13.34.

*Ethyl-6-amino-1,4-dihydro-4-(3-hydroxy-4-methoxyphenyl)-3-methylpyrano[2,3-c]pyrazole-5-carboxylate* **(5j)**. M.P. 145°C; IR (KBr): 3431, 3369, 3342, 3082, 2943, 1729, 1142, cm^−1^; ^1^H NMR (DMSO-d_6_): 1.32 (t, 3H, CH_3_), 2.77 (s, 3H, CH_3_), 3.71 (s, 3H, OCH_3_) 4.17 (q, 2H, CH_2_), 4.76 (s, 1H, CH), 5.22 (s, 1H, OH), 6.42–6.51 (m, 3H, ArH), 7.07 (s, 2H, NH_2_), 12.09 (s, 1H, NH), ppm. ^13^C NMR (400 MHz, DMSO): 10.34, 13.68, 38.86, 55.66, 61.82, 78.78, 115.36–132.38, 140.08, 148.44, 158.12, 160.32, 164.22 ppm. Anal. calcd for C_17_H_19_N_3_O_5_: C, 59.12; H, 5.55; N, 12.17. Found: C, 59.33; H, 5.57; N, 12.15.

## 4. Conclusion

We have demonstrated a highly efficient green catalytic approach for the four-component one-pot synthesis of pyranopyrazole derivatives catalyzed effectively by ZnO nanoparticles. ZnO nanoparticles are well characterized by XRD technique. This method offers several advantages including avoidance of harmful organic solvents, high yield, short reaction time, simple work-up procedure, ease of separation, and recyclability of the catalyst.

## Figures and Tables

**Table 1:** Nano-ZnO catalyzed synthesis of pyrano[2,3-*c*]pyrazole derivatives in water at room temperature **(5a–j)**.

Entry	Ar	Time (min.)	Yield (%)	M.P. (°C)
Ethyl-6-amino-1,4-dihydro-4-(3,4-dimethoxyphenyl)-3-methylpyrano[2,3-*c*]pyrazole-5-carboxylate **(5a)**	3,4-Dimethoxy phenyl	60	90	135

Ethyl-6-amino-1,4-dihydro-4-(3-methoxyphenyl)-3-methylpyrano[2,3-*c*]pyrazole-5-carboxylate **(5b)**	3-Methoxyphenyl	55	85	120

Ethyl-6-amino-1,4-dihydro-4-(3,4,5-trimethoxyphenyl)-3-methylpyrano[2,3-*c*]pyrazole-5-carboxylate **(5c)**	3,4,5-Trimethoxy phenyl	55	86	160

Ethyl-6-amino-4-(4-chlorophenyl)-1,4-dihydro-3-methylpyrano[2,3-*c*]pyrazole-5-carboxylate **(5d)**	4-Chlorophenyl	60	87	140

Ethyl-6-amino-1,4-dihydro-4-(4-methoxyphenyl)-3-methylpyrano[2,3-*c*]pyrazole-5-carboxylate **(5e)**	4-methoxyphenyl	55	89	130

Ethyl-6-amino-1,4-dihydro-3-methyl-4-(5-methylfuran-2-yl)pyrano[2,3-*c*]pyrazole-5-carboxylate **(5f)**	3-methyl-2-furyl	60	86	142

Ethyl-6-amino-1,4-dihydro-3-methyl-4-(thiophen-2-yl)pyrano[2,3-*c*]pyrazole-5-carboxylate **(5g)**	2-thienyl	55	87	115

Ethyl-6-amino-1,4-dihydro-3-methyl-4-(pyridin-3-yl)pyrano[2,3-*c*]pyrazole-5-carboxylate **(5h)**	3-pyridyl	60	85	125

Ethyl-6-amino-1,4-dihydro-4-(2-hydroxyphenyl)-3-methylpyrano[2,3-*c*]pyrazole-5-carboxylate **(5i)**	2-Hydroxyphenyl	60	87	143

Ethyl-6-amino-1,4-dihydro-4-(3-hydroxy-4-methoxy phenyl)-3-methylpyrano[2,3-*c*]pyrazole-5-carboxylate **(5j)**	3-hydroxy, 4-methoxyphenyl	60	85	145

Reaction conditions: hydrazine hydrate **(1)** (1 mmol), methyl acetoacetate **(2)** (1 mmol), substituted aromatic aldehyde **(3)** (1 mmol), ethylcyano acetate **(4)** (1 mmol), and ZnO nanoparticle (9 mol%) in water (2 mL).

**Table 2:** Screening of catalysts for one-pot condensation of ethyl cyanoacetate, hydrazine hydrate, 4-methoxy benzaldehyde, and methyl acetoacetate.

Entry	Catalyst	Catalyst (mol %)	Yield (%)	Time (min.)
**5e**	Alum	3	66	110
**5e**	ZnO nps	9	89	60
**5e**	Mont K10	7	75	80
**5e**	P_2_O_5_	5	68	110
**5e**	Acidic alumina	7	63	100
**5e**	Silica	12	69	100
**5e**	Mont KSF	7	58	90
**5e**	Glacial acetic acid	12	60	90

Reaction conditions: hydrazine hydrate **(1)** (1 mmol), methyl acetoacetate **(2)** (1 mmol), 4-methoxy benzaldehyde **(3)** (1 mmol), and ethylcyano acetate **(4)** (1 mmol) in water (2 mL).

**Table 3:** Effect of solvent on the reaction of ethyl cyanoacetate, hydrazine hydrate, 4-methoxy benzaldehyde, and methyl acetoacetate under stirring at room temperature.

Entry	Solvent	Time (min)	Yield (%)
**1**	Ethanol	90	62
**2**	Methanol	80	68
**3**	Water	60	89

Reaction conditions: hydrazine hydrate **(1)** (1 mmol), methyl acetoacetate **(2)** (1 mmol), 4-methoxy benzaldehyde **(3)** (1 mmol), ethylcyano acetate **(4)** (1 mmol), and ZnO nanoparticle (9 mol %).

**Table 4:** Comparison of catalytic activity of ZnO nanoparticles in the synthesis of compound **5e** by conventional (Δ) heating method and stirring at 25°C.

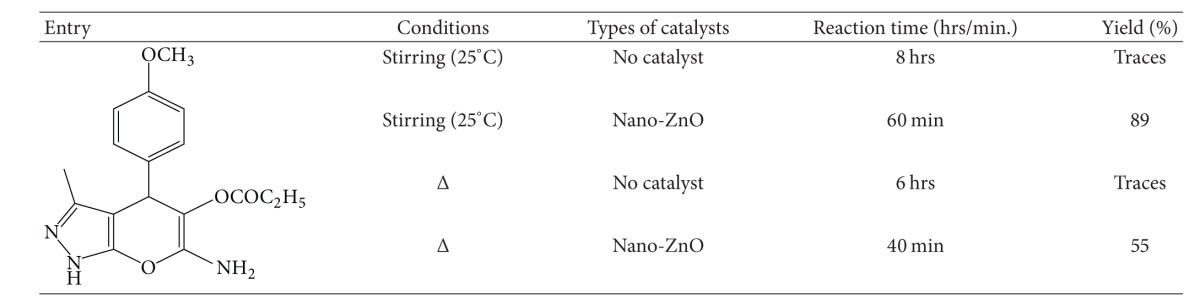

**Table 5:** Optimization of the ZnO nanoparticle catalyzed model reaction for synthesis of **5e**.

Entry	Catalyst (mol %)	Yield (%)
**1**	3	75
**2**	6	82
**3**	9	89

## References

[1] Orru RVA,  Greef M (2003). Recent advances in solution-phase multicomponent methodology for the synthesis of heterocyclic compounds. *Synthesis*.

[2] Paravidino M, Bon RS, Scheffelaar R (2006). Diastereoselective multicomponent synthesis of dihydropyridones with an isocyanide functionality. *Organic Letters*.

[3] Elders N, Schmitz RF, Kanter FJJ, Ruijter E, Groen MB, Orru RVA (2007). A resource-efficient and highly flexible procedure for a three-component synthesis of 2-imidazolines. *Journal of Organic Chemistry*.

[4] Groenendaal B, Vugts DJ, Schmitz RF (2008). A multicomponent synthesis of triazinane diones. *Journal of Organic Chemistry*.

[5] Groenendaal B, Ruijter E, Orru RVA (2008). 1-Azadienes in cycloaddition and multicomponent reactions towards N-heterocycles. *Chemical Communications*.

[6] Clark JH (2001). Catalysis for green chemistry. *Pure and Applied Chemistry*.

[7] Mohanraj VJ, Chen Y (2006). Nanoparticles: a review. *Tropical Journal of Pharmaceutical Research*.

[8] Astruc D (2007). Palladium nanoparticles as efficient green homogeneous and heterogeneous carbon-carbon coupling precatalysts: a unifying view. *Inorganic Chemistry*.

[9] Zhong L-S, Hu J-S, Cui Z-M, Wan L-J, Song W-G (2007). In-situ loading of noble metal nanoparticles on hydroxyl-group-rich titania precursor and their catalytic applications. *Chemistry of Materials*.

[10] Moreno-Mañas M, Pleixats R (2003). Formation of carbon-carbon bonds under catalysis by transition-metal nanoparticles. *Accounts of Chemical Research*.

[11] Astruc D (2008). *Nanoparticles and Catalysis*.

[12] Djakovitch L, Koehler K, Vries JG (2008). The role of palladium nanoparticles as catalysts for carbon-carbon coupling reactions. *Nanoparticles and Catalysis*.

[13] Durand J, Teuma E, Gómez M (2008). An overview of palladium nanocatalysts: surface and molecular reactivity. *European Journal of Inorganic Chemistry*.

[14] Kung HH (1989). *Transition Metal Oxides: Surface Chemistry and Catalysis*.

[15] Nohavica D, Gladkov P ZnO nanoparticles and their applications-new achievements.

[16] Moghaddam FM, Saeidian H, Mirjafary Z, Sadeghi A (2009). Rapid and efficient one-pot synthesis of 1,4-dihydropyridine and polyhydroquinoline derivatives through the hantzsch four component condensation by zinc oxide. *Journal of the Iranian Chemical Society*.

[17] Tamaddon F, Amrollahi MA, Sharafat L (2005). A green protocol for chemoselective O-acylation in the presence of zinc oxide as a heterogeneous, reusable and eco-friendly catalyst. *Tetrahedron Letters*.

[18] Kumar BV, Naik HSB, Girija D, Kumar BV (2011). ZnO nanoparticle as catalyst for efficient green one-pot synthesis of coumarins through Knoevenagel condensation. *Journal of Chemical Sciences*.

[19] Hosseini-Sarvari M (2011). Synthesis of quinolines using nano-flake ZnO as a new catalyst under solvent-free conditions. *Journal of the Iranian Chemical Society*.

[20] Yavari I, Beheshti S (2011). ZnO nanoparticles catalyzed efficient one-pot three-component synthesis of 2,3-disubstituted quinalolin-4(1H)-ones under solvent-free conditions. *Journal of the Iranian Chemical Society*.

[21] Alinezhad H, Salehian F, Biparva P (2012). Synthesis of benzimidazole derivatives using heterogeneous ZnO nanoparticles. *Synthetic Communications*.

[22] Goda FE, Maarouf AR, EL-Bendary ER (2003). Synthesis and antimicrobial evaluation of new isoxazole and pyrazole derivatives. *Saudi Pharmaceutical Journal*.

[23] El-Emary TI (2006). Synthesis and biological activity of some new pyrazolo[3,4-b]pyrazines. *Journal of the Chinese Chemical Society*.

[24] Mansour AK, Eid MM, Khalil NSAM (2003). Synthesis and reactions of some new heterocyclic carbohydrazides and related compounds as potential anticancer agents. *Molecules*.

[25] Abunada NM, Hassaneen HM, Kandile NG, Miqdad OA (2008). Synthesis and antimicrobial activity of some new pyrazole, fused pyrazolo[3,4-d]-pyrimidine and pyrazolo[4,3-e][1,2,4]-triazolo[1,5-c]pyrimidine derivatives. *Molecules*.

[26] Ranatunge RR, Garvey DS, Janero DR (2004). Synthesis and selective cyclooxygenase-2 (COX-2) inhibitory activity of a series of novel bicyclic pyrazoles. *Bioorganic and Medicinal Chemistry*.

[27] Bekhit AA, Ashour HMA, Abdel Ghany YS, Bekhit AE-DA, Baraka A (2008). Synthesis and biological evaluation of some thiazolyl and thiadiazolyl derivatives of 1H-pyrazole as anti-inflammatory antimicrobial agents. *European Journal of Medicinal Chemistry*.

[28] Fioravanti R, Bolasco A, Manna F (2010). Synthesis and biological evaluation of N-substituted-3,5-diphenyl-2- pyrazoline derivatives as cyclooxygenase (COX-2) inhibitors. *European Journal of Medicinal Chemistry*.

[29] Kuo S-C, Huang L-J, Nakamura H (1984). Synthesis and analgesic and antiinflammatory activities of 3,4-dimethylpyrano[2,3-c]pyrazol-6-one derivatives. *Journal of Medicinal Chemistry*.

[30] Vaghasiya SJ, Dodiya DK, Trivedi AR, Shah VH (2008). Synthesis and biological screening of some novel pyrazolo[3′, 4′:4,5]thieno[2,3-d]pyrimidin-8-ones via a Gewald reaction. *Arkivoc*.

[31] Abdelrazek FM, Metz P, Metwally NH, El-Mahrouky SF (2006). Synthesis and molluscicidal activity of new cinnoline and pyrano[2,3-c]pyrazole derivatives. *Archiv der Pharmazie*.

[32] Harshad G, Kathrotiya R, Patel R, Patel MP (2012). Microwave-assisted multi-component synthesis of indol-3-yl substituted pyrano[2, 3-c]pyrazoles and their antimicrobial activity. *Journal of the Serbian Chemical Society*.

[33] Madhusudana Reddy MB, Pasha MA (2012). One-pot, multicomponent synthesis of 4H-pyrano[2,3-c]pyrazoles in water at 25°C. *Indian Journal of Chemistry B*.

[34] Shinde PV, Gujar JB, Shingate BB, Shingare MS (2012). Silica in water: a potentially valuable reaction medium for the synthesis of pyrano[2,3-c]pyrazoles. *Bulletin of the Korean Chemical Society*.

[35] Heravi MM, Ghods A, Derikvand F, Bakhtiari K, Bamoharram FF (2010). H14[NaP5W30O110] catalyzed one-pot three-component synthesis of dihydropyrano[2,3-c]pyrazole and pyrano[2,3-d]pyrimidine derivatives. *Journal of the Iranian Chemical Society*.

[36] Mecadon H, Rohman MR, Kharbangar I (2011). L-Proline as an efficicent catalyst for the multi-component synthesis of 6-amino-4-alkyl/aryl-3-methyl-2,4-dihydropyrano[2,3-c]pyrazole-5-carbonitriles in water. *Tetrahedron Letters*.

[37] Darandale SN, Sangshetti JN, Shinde DB (2012). Ultrasound mediated sodium bisulfite catalyzed solvent-free synthesis of 6-amino-3-methyl-4-substitued-2,4-dihydropyrano[2, 3-c]pyrazole-5-carbonitrile. *Journal of the Korean Chemical Society*.

[38] Chavan HV, Babar SB, Hoval RU, Bandgar BP (2011). Rapid one-pot, four component synthesis of pyranopyrazoles using heteropolyacid under solvent-free condition. *Bulletin of the Korean Chemical Society*.

[39] Tekale SU, Kauthale SS, Jadhav KM, Pawar RP (2013). Nano-ZnO catalyzed green and efficient one-pot four-component synthesis of pyranopyrazoles. *Journal of Chemistry*.

[40] Safaei-Ghomi J, Ziarati A, Tamimi M (2013). A Novel method for the one-pot five-component synthesis of highly functionalized pyranopyrazoles catalyzed by CuI nanoparticles. *Acta Chimica Slovenica*.

[41] Myrboh B, Mecadon H, Rohman R (2013). Synthetic developments in functionalized pyrano[2, 3-c]pyrazoles. A review. *Organic Preparations and Procedures International*.

[42] Vasuki G, Kumaravel K (2008). Rapid four-component reactions in water: synthesis of pyranopyrazoles. *Tetrahedron Letters*.

[43] Wu M, Feng Q, Wan D, Ma J (2013). CTACl as catalyst for four-component, one-pot synthesis of pyranopyrazole derivatives in aqueous medium. *Synthetic Communications*.

[44] Bihani M, Bora PP, Bez G (2013). Practical catalyst-free synthesis of 6-amino-4 alkyl/aryl-3-methyl-2,4-dihydropyrano[2,3-c]pyrazole-carbonitrile in aqueous medium. *Journal of Chemistry*.

[45] Sachdeva H, Dwivedi D (2012). Lithium-acetate-mediated biginelli one-pot multicomponent synthesis under solvent-free conditions and cytotoxic activity against the human lung cancer cell line A549 and breast cancer cell line MCF7. *The Scientific World Journal*.

[46] Sachdeva H, Dwivedi D, Bhattacharjee RR, Khaturia S, Saroj R (2013). NiO nanoparticles: an efficient catalyst for the multicomponent one-pot synthesis of novel spiro and condensed indole derivatives. *Journal of Chemistry*.

[47] Dandia A, Sachdeva H, Singh R (2001). Improved synthesis of 3-spiro indolines in dry media under microwave irradiation. *Synthetic Communications*.

[48] Pacholski C, Kornowski A, Weller H (2002). Self-assembly of ZnO: from nanodots to nanorods. *Angewandte Chemie*.

